# The skin through reflectance confocal microscopy — Historical background, technical principles, and its correlation with histopathology^☆^

**DOI:** 10.1016/j.abd.2021.10.010

**Published:** 2022-09-21

**Authors:** Naiara Fraga Braghiroli, Samantha Sugerik, Luiz Antônio Rodrigues de Freitas, Margaret Oliviero, Harold Rabinovitz

**Affiliations:** a Dermatology Department, Miami Cancer Institute, Miami, FL, United States; b Department of Human Pathology, Oswaldo Cruz Foundation, Salvador, BA, Brazil; c Medical School, Florida Atlantic University College of Medicine, BocaRaton, FL, United States; d Department of Pathology, Federal University of Bahia, Salvador, BA, Brazil; e Dermatology Department, Skin Cancer & Associates, Plantation, FL, United States

**Keywords:** Microscopy, confocal, Pathology, Skin neoplasm, Skin abnormalities

## Abstract

Since its first introduction into medical practice, reflectance confocal microscopy (RCM) has been a valuable non-invasive diagnostic tool for the assessment of benign and malignant neoplasms of the skin. It has also been used as an adjunct for diagnosing equivocal cutaneous neoplasms that lack characteristic clinical or dermoscopic features. The use of RCM has led to a decreased number of biopsies of benign lesions. Multiple published studies show a strong correlation between RCM and histopathology thereby creating a bridge between clinical aspects, dermoscopy, and histopathology. Dermatopathologists may potentially play an important role in the interpretation of confocal images, by their ability to correlate histopathologic findings. RCM has also been shown to be an important adjunct to delineating tumoral margins during surgery, as well as for monitoring the non-surgical treatment of skin cancers. Advanced technology with smaller probes, such as the VivaScope 3000, has allowed access to lesions in previously inaccessible anatomic locations. This review explains the technical principles of RCM and describes the most common RCM features of normal skin with their corresponding histological correlation.

## Introduction

Since its introduction into clinical practice, Reflectance Confocal Microscopy (RCM) has been valuable in the noninvasive diagnosis of benign and malignant neoplasms of the skin. It has been used as an ancillary tool in lesions with equivocal clinical and dermoscopic features. Thus, RCM has reduced the necessity of performing biopsies of benign lesions, particularly in aesthetically sensitive areas.

RCM is a non-invasive imaging technique that provides cellular detail from the epidermis down to the papillary dermis. In contrast to conventional histopathology, sections are viewed horizontally to the surface of the skin. The correlation between RCM's findings and histology allows for the inclusion of the dermatopathologist as an important collaborator in the evaluation and interpretation of images acquired with the RCM.

This paper presents a review of the historical and technical aspects of the RCM, as well as provides a brief review of the findings of normal skin through the lens of the confocal microscope.

## Historical background

The technology of the reflectance confocal microscope was created in 1955, by Marvin Minsky, and patented in 1957. Confocal microscopy provided the capacity for direct, noninvasive, serial optical sectioning of intact skin, with instant viewing of zoomed images with high resolution, in the horizontal plane, while moving the point under the illumination of the object under examination.^1^

The first scientific publication with data and images generated by a confocal microscope was authored by M. David Eggar and Petran, in 1967; the authors describe the project and the construction of the confocal microscope. In 1969, and later in 1971, Davidovits and Egger published the first studies using the confocal microscope with laser scanning in the evaluation of organic tissues, using nerve fragments *ex vivo*.^2^, ^3^

The analysis of living tissue, however, was initiated in 1980, when several research groups developed the first studies on material from human organisms and other animals. The mapping of human skin with the use of the RCM was performed in 1995, by Rajadhyaksha et al., pioneers in obtaining instantaneous and non-destructive high-resolution images of the skin.^4^

## Technical principles

Since 1995, the use of reflectance confocal microscopy has increased in major dermatologic academic centers around the world. With advancements in technology and subsequent improvements in functionality and design, the device has helped to revolutionize the approach to diagnosis and management of cutaneous neoplasms.

The principle of RCM involves the use of a spotlight source that illuminates a focal point in the skin. The reflected image is then displayed in a detector after passing through a small hole, allowing only the area in focus to be detected. The RCM's technology is based on the use of a low-power laser (diode laser), which emits infra-red light of 830 nanometers (nm). The optical section of the images of the RCM is comparable to the resolution of the histopathological images of 30×. There are two types of RCM equipment currently available for the diagnosis of skin lesions. The traditional model (VivaScope 1500; CaliberID, Rochester, NY) composes the optical sections in a mosaic of images, allowing a field of view of 838 mm^2^. The portable model (Vivascope 3000; CaliberID, Rochester, NY), introduced into dermatological practice in 2011, has a smaller probe that allows the assessment of lesions in curved areas of the face and anatomic areas that are difficult to access. This model provides a limited field of view of 131 mm^2^.

The Vivascope 1500 model provides a lateral resolution of about 1 μm and an axial resolution of 3–5 μm. It reaches a depth of about 200–250 μm, allowing the visualization of the papillary dermis. In areas with the thinner epidermis, such as the face and mucous membrane, it is possible to analyze the superficial reticular dermis as well. With this model, a metal ring with a plastic window is placed over the lesion. Immersion oil acts as an interface between the skin and the plastic ring, and ultrasound gel is the medium in which the lens is immersed prior to coupling. The RCM scans the skin in the horizontal direction, producing multiple individual images of 0.5 × 0.5 mm^2^, forming a mosaic of up to 8 × 8 mm^2^. The precise depth is controlled by the navigation software and selected by the examiner, up to a maximum depth of 250 micrometers. The operator navigates the system guided by the dermatoscopic picture of the lesion, which was obtained after the placement of the fixation ring and before attaching the lens. This allows for orientation and systematic imaging of the lesion.^5^, ^6^

The Vivascope 3000, is a model with a portable probe easy to mobilize. The advantage of this model is its’ small probe, allowing access to hard-to-reach areas. The process of obtaining images is faster with the Vivascope 3000. The disadvantage is the small field of view 1 × 1 mm^2^ and the ability to take only vertical as opposed to horizontal sections. The handheld RCM model has been proven particularly useful in the diagnosis of solitary facial papules, with a sensitivity of 93% and specificity of 78% in the diagnosis of basal cell carcinoma (BCC).^6^, ^7^

### Clinical applications

The reflectance confocal microscope has the advantage of allowing real-time, non-invasive “virtual-histologic” assessment of the neoplasm.

The device allows for the assessment of more of the lesion, in contrast to histopathology, in which only approximately 2% of the specimen is analyzed. The images obtained by RCM can therefore potentially define tumor margins during surgical resections. For instance, some studies have shown it to be a valuable adjunct in the definition of surgical margins during Mohs micrographic surgery. Studies have demonstrated a sensitivity of 86%–96% and specificity of 89%–99% in the identification of BCC during Mohs surgery.^8^, ^9^, ^10^

The reflectance confocal microscope has been used as an ancillary tool in lesions with equivocal clinical and dermatoscopic features, increasing the accuracy in the selection of neoplasms that require biopsy.^6^, ^8^

Another important indication of confocal microscopy is in monitoring the response to non-surgical treatment of actinic keratosis, squamous cell carcinoma (SCC), BCC, and melanoma in photo-exposed areas.^11^, ^12^, ^13^, ^14^, ^15^

## RCM features of normal skin and its correlation with histology

As previously stated, there is an excellent correlation between histology and RCM, with RCM as a bridge between clinical, dermoscopy, and histopathology.

The visualization of structures with confocal microscopy is based on the difference in the refraction of light. Structures with a high refractive index appear shiny and white, while structures with a low refractive index appear black. Melanin as a high contrast agent, with a refractive index of 1.7, allows for the recognition of individual cells rich in melanin, like the melanocytes, pigmented keratinocytes, and melanophages. Keratin has a refractive index of 1.5, allowing the visualization of neoplasms of keratinocytes.

The collagen fibers and inflammatory cells, such as neutrophils, with a refractive index of 1.34, also appear shiny in the images obtained with the RCM. The collagen fibers appear as linear shiny bands in the dermis, and the inflammatory cells as small, white, rounded, and homogeneous structures. Inflammatory cells, despite having a refractive index lower than melanin, still appear as shiny particles at RCM.^5^, ^6^ However, the majority of inflammatory diseases of the skin studied using the RCM technology were lesions rich in lymphocytes. The criteria for differentiation between lymphocytes and neutrophils have not been established.^16^, ^17^, ^18^

### Stratum corneum

**Figure 1:**
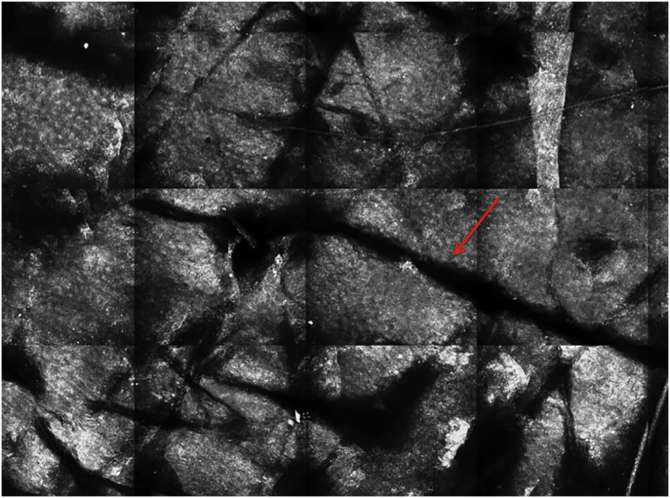
RCM of the *stratum corneum*. (A) Flat enucleated polygonal cells represent the corneocytes, linear dark structures representing the dermatoglyphics (red arrow).

**Figure 2:**
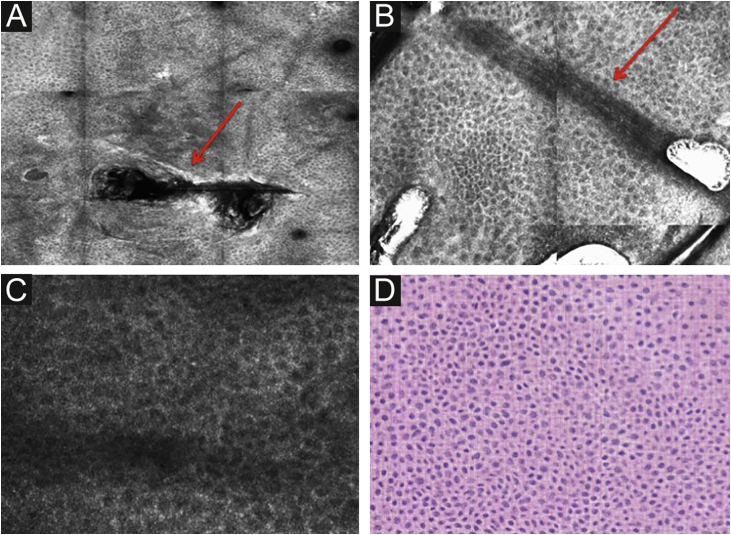
RCM of the spinous-granular layer. (A) Typical honeycomb pattern; amorphous hyper-reflective structure representing the scale (red arrow) (B) Typical honeycomb pattern, linear dark structures representing the dermatoglyphics (red arrow). (C) Higher magnification RCM image showing a typical honeycomb pattern, characterized by monomorphic polygonal cells with a thin outlier representing the typical keratinocytes. (D) Histopathology: Typical keratinocytes with uniformity of size and shape (Hematoxylin & eosin ×400).

The first layer of skin to be evaluated is the *stratum corneum*. At this level, the flattened cells without nuclei, that aggregate forming islands separated by dark cracks, can be appreciated (Fig. 1). The linear dark structures visualized represent the dermatoglyphics of the skin. The cells correspond to the anucleate corneocytes that stand out in confocal microscopy, due to their high content of keratin. At this level, the scales are visualized as amorphous highly reflective structures (Fig. 2A and B).^5^, ^16^

### Spinous-granular and basal layer

Below the *stratum corneum*, approximately 15–20 μm in depth, is the granular layer. At this level, the keratinocytes are flattened, with internal granules, characterized in confocal by central dark nuclei surrounded by the white glossy contour of the cytoplasm. This is due to the keratin granules located therein. The layout of the keratinocytes adjacent to each other in this layer follows a pattern known as typical or regular “honeycomb” (Fig. 2C).

The spinous layer is located between 20–100 μm in depth. It appears on RCM with similar morphology to the granular layer, with thetypical honeycomb pattern, but with smaller keratinocytes.

**Figure 3:**
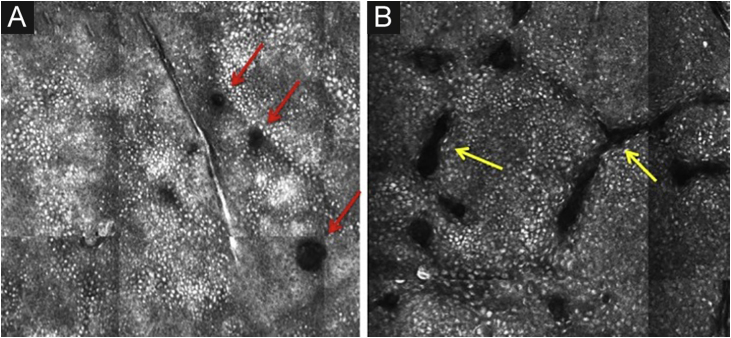
RCM of the basal layer. (A and B) Cobblestone pattern, characterized by sets of white shiny and small round structures representing the pigmented basal keratinocytes. Dark round structures represent the hair follicles (red arrows) and dark linear fissures representing the invaginations of the skin (yellow arrows).

The basal layer is located between 40 and 130 μm in-depth and its appearance on RCM varies according to the skin phototype. Phototypes III‒IV skin displays a pattern known as cobblestone, which is formed by sets of white shiny and small keratinocytes (Fig. 3). The high reflectivity of these cells is due to the supranuclear melanin cap, providing high luminance around the nucleus, in contrast with the rest of the cytoplasm that is relatively darker. The basal keratinocytes in phototype I‒II skin are less pigmented; therefore these cells do not appear as shiny in confocal microscopy.^5^, ^16^

### Dermal-epidermal junction and papillary dermis

**Figure 4:**
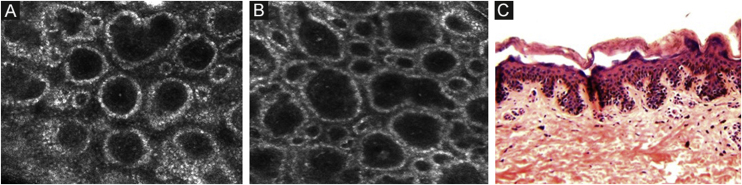
RCM of the Dermal-Epidermal Junction (DEJ). (A and B) Edge papillae formed by well demarcated papillary rings around a dark center representing the pigmented keratinocytes and/or basal melanocytes surrounding dark dermal papilla. *The ringed pattern* forms the edge papillae. (C) Histologically, the ringed pattern represents elongated rete-ridges with an increased number of single melanocytes at the basal layer.

The melanocytes in the Dermal-Epidermal Junction (DEJ) are not usually identifiable in normal skin from individuals with phototype I‒II skin. The DEJ with RCM is characterized by bright basal keratinocytes surrounding dark dermal papilla, creating a pattern known as edged papillae, formed by well-demarcated papillary rings around a dark center (Fig. 4).

The papillary and superficial reticular dermis appear with low reflectivity and may display blood vessels and collagen fibers.^5^, ^16^

**Figure 5:**
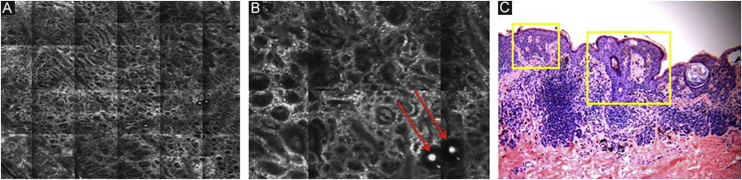
RCM of the Dermal-Epidermal Junction (DEJ). (A) *Meshwork pattern*, characterized by elongated hyper-reflective cords, corresponding the junctional thickening. (B) Higher magnifications showing enlongated cords interconnecting, round white structures represent the milia-like cysts (red arrows). (C) Histopathology showing the enlargement of the interpapillary space, formed by aggregated melanocytes, with predominantly small, interconnecting nests at the tip of the rete ridges.

Pigmented melanocytic lesions can present with two patterns at the DEJ: the *ringed pattern*, characterized by a rim of bright cells forming the edged papillae (Fig. 4); and the *meshwork pattern*, characterized by elongated cords and junctional thickening (Fig. 5). Histologically, the ringed pattern represents elongated rete-ridges with an increased number of single melanocytes at the basal layer. The pigmented basal keratinocytes correspond to a lentiginous pattern. In contrast, the meshwork pattern corresponds in histopathology to the enlargement of the interpapillary space formed by aggregated melanocytes, with predominantly small, non-confluent nests at the tip of the rete ridges.

**Figure 6:**
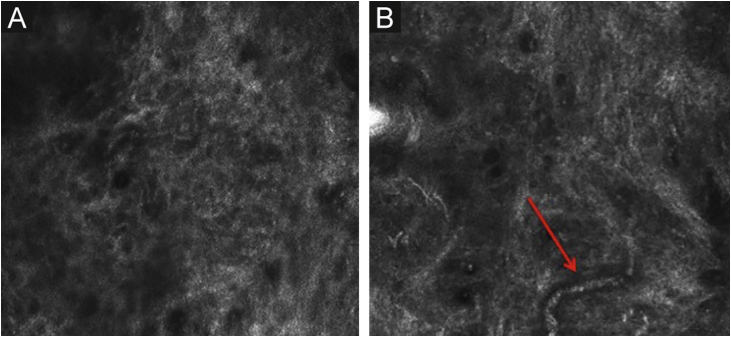
RCM of the papillary dermis. (A) Diffuse dark areas, with hyper reflective coils and parallel structures corresponding to the fibrillar collagen bundles. (B) The vessels appear as tubular dark structures with small shiny white structures corresponding to the blood cells (red arrow).

The papillary and superficial reticular dermis display blood vessels and collagen fibers. RCM images portray diffuse dark areas, with thin hyper-reflective coils and parallel structures at the papillary dermis (Fig. 6A), and white coarse structures at the upper reticular dermis. The white structures represent the collagen fibers. The vessels appear as tubular dark structures (Fig. 6B). Movement of blood cells through the vessels may be visualized during imaging.

The appearance of “normal skin” may vary depending on anatomic location, as well as the age of the patient, and the amount of cutaneous damage due to chronic sun exposure. Table 1 resumes the main RCM features of normal skin and their correlation with histology.

**Table 1:** RCM features of normal skin and correlation with histology.

	RCM features	Histology description
***Stratum corneum***	Flattened cells without nuclei that aggregate forming islands separated by dark cracks.	Enucleated corneocytes adjacent to each other.
	Amorphous highly reflective structures.	Scales

**Spinous-granular layer**	*Honeycomb pattern*, formed by round structures adjacent to each other with central dark nuclei surrounded by a white glossy contour of the cytoplasm, due to the keratin granules content.	Flat keratinocytes, with internal granules

**Basal layer**	*Cobblestone pattern*, formed by set of white shiny and small keratinocytes.	Small, pigmented keratinocytes.

**Dermal-epidermal junction**	Edge papillae, are characterized by well-demarcated rings around a dark center.	Bright basal keratinocytes surrounding the dark dermal papilla.
	Pigmented melanocytic lesions: *ringed pattern*, characterized by a rim of bright cells forming the edge papillae; *meshwork pattern*, characterized by junctional thickening.	*Ringed pattern*: elongated rete-ridges with an increased number of single melanocytes at the basal layer and pigmented basal keratinocytes corresponding to a lentiginous pattern.
		*Meshwork pattern*: enlargement of the interpapillary space formed by aggregated melanocytes, with predominantly small, non-confluent nest at the tip of the rete ridges.

**Papillary dermis**	Diffuse dark area with thin linear shiny bands at papillary dermis and white coarse structures at the upper reticular dermis. The vessels appear as tubular dark structures with small round white cells.	The white structures represent the collagen fiber in the dermis.
		Blood vessels and lymphocytes within blood the vessels.

## Limitations of the Reflectance Confocal Microscopy and future perspectives

One limitation of RCM is the inability to analyze structures below 250 microns in depth. Consequently, alterations in the reticular dermis and invasive tumors cannot be evaluated. In lesions with significant thickening of the epidermis, including acral lesions, the examination with the confocal is limited to the epidermis.

There are factors that also may compromise the quality of the image. Hyperkeratosis and residues of creams containing particles with a high refraction index (i.e., sunscreen) may create artifacts. The non-uniform surface of the skin also may result in the formation of bubbles, which constitute artifacts.

The interpretation of the RCM images requires training. Robust knowledge of dermatopathology is an asset, as RCM structures correlate with histologic findings. There is the challenge of distinguishing structures and cells with similar refraction index, (i.e., melanocytes and Langerhans cells in the stratum *spinosum*).

Studies have shown the reflectance confocal microscope as an ancillary tool in the diagnosis of infectious and inflammatory skin diseases, such as psoriasis, contact dermatitis, cutaneous T-cell lymphoma, discoid lupus erythematosus, and onychomycosis. However, there is a lack of studies with appropriate methodology demonstrating the ability to distinguish different types of inflammatory cells with confocal.^4^, ^17^, ^18^

A series of new projects in the context of technological development seems promising for the future use of the RCM. Devices that capture images of the skin using an in-line scan, instead of scanning a point, are already being developed. This will reduce the time of image acquisition. Confocal microscopy machines with software programmed for the automated recognition of anatomic details of the skin are also being studied, with the possibility of reconstruction of images in 3 dimensions. 19, 20

Finally, fluorescent confocal images that appear after intradermal injection of contrast in vivo, as indocyanine green, are also in the testing phase, with the aim of improving the identification of the structures viewed with the RCM. 21, 22

## Conclusions

With nearly 20 years of use in dermatological practice, the reflectance confocal microscope has proven to be an effective diagnostic adjunct in the diagnosis of a variety of neoplasms of the skin. Certainly, its use has aided in the management of clinically equivocal lesions, reducing biopsies, and thereby cost, as well as patients’ anxiety over more invasive diagnostic procedures. In the near future, with advanced technology, RCM has the potential to provide more detailed information in a shorter period of time, to assist the physician in the diagnosis and management of patients.

## Financial support

None declared.

## Authors’ contributions

Naiara Fraga Braghiroli made substantial contributions to the conception and design of the manuscript, acquisition, analysis and interpretation of data; read and approved the final manuscript.

Samantha Sugerik had been involved in the revision, formatting, and submission of the manuscript; read and approved the final manuscript.

Luiz Antônio Rodrigues de Freitas reviewed the histologic digital images, reviewed the final manuscript and gave the final approval of the version to be published; read and approved the final manuscript.

Margaret Oliviero Had been involved in the acquisition, analysis and interpretation of data, as well as drafting the manuscript and revising it critically for important intellectual content; read and approved the final manuscript.

Harold Rabinovitz had been involved in the analysis and interpretation of data, he also reviewed the final manuscript and gave final approval of the version to be published; read and approved the final manuscript.

## Conflict of interest

None declared.
